# High‐energy diet improves growth performance, meat quality and gene expression related to intramuscular fat deposition in finishing yaks raised by barn feeding

**DOI:** 10.1002/vms3.306

**Published:** 2020-06-25

**Authors:** Kun Kang, Jian Ma, Hongze Wang, Zhisheng Wang, Quanhui Peng, Rui Hu, Huawei Zou, Shanke Bao, Wenhua Zhang, Baozhong Sun

**Affiliations:** ^1^ Key Laboratory of Low Carbon Culture and Safety Production in Cattle in Sichuan Animal Nutrition Institute of Sichuan Agricultural University Chengdu China; ^2^ Haibei Demonstration Zone of Plateau Modern Ecological Animal Husbandry Science and Technology Haibei China; ^3^ Ningxia Xiahua Meat Product Limited Company Zhongwei China; ^4^ Institute of Animal Science Chinese Academy of Agricultural Sciences Beijing China

**Keywords:** energy concentration, finishing yaks, growth performance, intramuscular fat, meat quality

## Abstract

This research aimed to investigate the effects of dietary energy concentration (combined net energy, Nemf) on growth performance and meat quality of yaks raised by barn feeding. In all, 30 male yaks (3‐year old and 114.57 ± 21.56 kg of body weight) were allocated to one of three isonitrogenous dietary treatments that had different Nemf concentrations (low 3.72 MJ/kg, middle 4.52 MJ/kg and high 5.32 MJ/kg, respectively). The yaks were fed for 120 days. The results showed that the final weight, average daily gain, dressing percentage, backfat thickness and loin muscle area were significantly improved (*p* < .05) with the increase in dietary energy concentration. However, an opposite trend of feed:gain ratio, cooking loss, driage, shear force and moisture content was found. A significant improvement (*p* < .05) of intramuscular fat content was observed in the high‐energy group. Additionally, the proportion of polyunsaturated fatty acid was increased (*p* < .05) at the expense of the saturated fatty acids. The mRNA expressions of lipogenic genes fatty acid synthase, acetyl‐CoA carboxylase, sterol regulatory element‐binding protein 1, stearoyl‐CoA desaturase, peroxisome proliferator‐activated receptor γ, lipoprotein lipase and heart fatty acid‐binding proteins increased (*p* < .05) in a dose‐dependent manner. However, the mRNA expressions of lipolytic genes carnitine palmitoyltransferase‐1 and hormone‐sensitive lipase correspondingly decreased (*p* < .05) with increased dietary energy level. In summary, the growth performance, meat production and meat quality improvement of finishing yaks can be achieved by increasing the dietary energy concentration. The intramuscular fat accumulation of yaks was achieved through up‐regulation of intramuscular lipogenic gene expression as well as fatty acid transport gene expression and down‐regulation of lipolytic gene expression by promoting dietary energy concentration.

## Graphical abstract

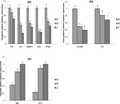

## 1 INTRODUCTION

Yaks (*Bos grunniens*) live in extremely harsh conditions at altitudes from 2000 m to 5,000 m above sea level (Guan et al., 2017). More than 90% of the world's total yak population inhabits the Qinghai‐Tibetan Plateau in China, where milk and meat are the major food and financial income for local Tibetan herders (Hu et al., 2019). However, due to the special geographical environment of the Qinghai‐Tibetan Plateau, under the traditional farming system, yaks inevitably suffer from insufficient feeding resources in the long cold season (October–May). The long slaughter cycle (usually 9 years) results in low production of meat and a poor meat quality (Han, Xie, Bi, Liu, & Hu, 2002; Wan et al., 2011). Previous research has reported that the meat of grazing yaks contain favourable amino acid and fatty acid profiles as well as a high protein content, but after cooking, the meat tenderness is poor (Luo, Tong, Wei, & Zhao, 2006). Therefore, yak meat is mostly used to make beef jerky, which limits its development and utilization. With the global rise of the importance of green food and growing consumption demand, there is much concern directed towards the production of high‐quality yak meat domestically as well as abroad. Hence, it is an urgent problem to improve quality of yak meat through nutritional strategies.

Previous study has reported that in the yak farming system, feed supplementation regimes can reduce the weight loss (Long, Dong, Wei, & Pu, 2005). Moreover, dietary energy concentration has positive effects on growth performance, carcass traits and meat quality of livestock (Long et al., 2004; Zhang, Wang, Peng, Tan, & Zou, 2014). Previous study has reported that yaks raised in warming sheds show a higher apparent digestibility and average daily gain (ADG) with the increased dietary energy level during the winter in the Tibetan plateau (Dong, Zhao, Ma, Xu, & Li, 2006). However, the effects of dietary energy concentration on carcass traits and meat quality of yaks raised by barn feeding have not been fully evaluated.

The juiciness, flavour, tenderness and overall quality of meat are significantly associated with intramuscular fat (IMF) content and fatty acid profile (Anton et al., 2018; O’Quinn et al., 2012). However, the IMF content of yak meat is low, which is one of the main factors that limit its acceptance by consumers. The IMF content is influenced by a number of factors, including age, gender, breed and nutrition (Maltin, Balcerzak, Tilley, & Delday, 2003). Studies found that increased dietary energy concentration could promote IMF content, resulting in improved meat quality (Cromwell, Hays, Trujillo‐Fig ueroa, & Kemp, 1978; Liu et al., 2007). Based on the previous studies, we hypothesized that the dietary energy concentration might improve the meat quality of finishing yaks raised by barn feeding. Therefore, the aim of this study was to determine the effects of dietary energy concentration on growth performance, meat quality and gene expression related to IMF deposition of yaks that were raised indoor.

## 2 MATERIALS AND METHODS

### 2.1 Animals, experimental diets and design

The experiments were carried out from January to July of 2018 at the Plateau farm. A total of 30 Qinghai plateau male yak (3‐year old and 114.57 ± 21.56 kg of body weight (BW)) were selected and randomly assigned to three groups: low energy (LE, 3.72 MJ/kg, Nemf), middle energy (ME, 4.52 MJ/kg, Nemf) and high energy (HE, 5.32 MJ/kg, Nemf). All yaks were housed in 15 pens according to corresponding group, with 2 yaks in each pen (4 × 4 m). Each pen also had a fenced area used as a playground for the yaks during daytime.

In the current study, the basal diets were formulated according to the Chinese Beef Cattle Raising Standard (NY/T 815, 2004) for finishing beef cattle, and the nutrient levels of basal diets were fully met or exceeded the recommended nutrient requirements. Additionally, all the experimental diets were isonitrogenous. The ratio of the ME group was designed according to the nutrient requirements of 150 kg finishing beef cattle with an ADG of 800 g. Compared with the ME group, the dietary energy concentration of the HE and LE group changed by 0.8 MJ/kg. The ratio of roughage to concentrate in the diet was 70:30, and the feed compositions and nutrient levels of the experimental diets are described in Table 1. All the yaks were fed with the total mixed ration, and yaks were submitted to 30 days of acclimatization to experimental installations and diets followed by 120 days of the formal experiment. During the experiment, all yaks were fed twice daily at 08:30 and 17:00, and they had ad libitum access to ration and water.

**TABLE 1:** Composition and nutrient levels of experimental rations (Air‐dry basis, %)

Items	Treatments^1^		
	LE	ME	HE
Corn (%)	2.20	15.84	22.75
Wheat bran (%)	22.03	6.62	0.40
Rapeseed meal (%)	1.25	2.25	1.10
Soybean meal (%)	2.95	3.38	3.37
Calcium hydrogen carbonate (%)	0	0.59	1.20
Limestone (%)	0.64	0.39	0.25
Sodium bicarbonate (%)	0.30	0.30	0.30
Salt (%)	0.30	0.30	0.30
Choline chloride (%)	0.03	0.03	0.03
Premix^2^ (%)	0.30	0.30	0.30
Oats hay (%)	60.00	47.50	30.00
Distilled grain (%)	10.00	22.50	40.00
Nutrient level^3^			
Nemf^4^(MJ/kg)	3.72	4.52	5.32
Crude protein (%)	12.57	12.57	12.57
Neutral detergent fibre (%)	47.64	43.43	41.19
Acid detergent fibre (%)	26.27	25.34	24.58
Crude fat (%)	4.81	5.56	6.43
Calcium (%)	0.60	0.59	0.60
Phosphorus (%)	0.40	0.39	0.40

^1^ LE, low energy; ME, medium energy; HE, high energy.

^2^ Premix was formulated to provide the following per kg of total diet DM: 600 IU of vitamin A, 275 IU of vitamin D, 60 IU of vitamin E, 0.1 mg of Co, 10 mg of Cu, 0.5 mg of I, 30 mg of Mn, 0.2 mg of Se, 30 mg of Zn, 50 mg of Fe, and 20 mg of monensin.

^3^ All nutrient levels are calculated.

^4^ Nemf, combined net energy.

### 2.2 Growth performance

The BW of all yaks was measured on d 0 and 120 before the morning feeding. The ADG was calculated by the initial and final BW. Accurate average daily feed intake (ADFI) for each pen was recorded daily during the formal experiment. Feed efficiency (F/G, feed intake to gain ration) was determined by dividing ADFI by ADG.

### 2.3 Slaughter surveys and sampling

At the end of the trial, after fasted 12 hr, six yaks that were close to the group average weight from each treatment were moved to slaughter house. After obtaining the before slaughter liveweight, yaks were stunned using electricity, slaughtered by exsanguination, skinned, eviscerated and split down the middle according to the standard commercial procedures. Hot carcass weight was measured, and the testicles, kidneys and pelvic fat were maintained, then dressing percentage was calculated. Subsequently, samples of roughly 500 g of the *longissimus thoracis* muscle (LM) at the 12th–13th rib were immediately collected from the left side of the carcass, weighed, put in the sterile vacuum package and then stored at 4°C for meat quality determination. LM samples for fatty acid analysis and RNA extraction were rapidly removed and frozen in liquid nitrogen, and then stored in a refrigerator at −80°C. Fat depth opposite to the first rib, last rib and last lumbar vertebra were measured to calculate average backfat thickness. The cross‐sectional area of the LM in the left side of the carcass was measured at the 10th rib by planimetry. After cooling for 24 hr at 4°C, the carcasses were segmented and the primal cuts were weighed. Primal cuts were dissected and named according to the Chinese beef carcass and cuts standard (GB/T 27643–2011) after trimming. Based on the most popular and economic meat cuts in the market, the high rib, ribeye, striploin, tenderloin, brisket, shank, shoulder chops, outside flat, eyeround, topside and knuckles were considered as the primal cuts.

### 2.4 Meat quality

The pH value of the LM was determined at 45 min and 24 hr postmortem using a pH meter probe (PH200, Ruizhen Electronic Technology Co., LTD., Shanghai, China). The determination of meat colour was performed as described by Houben, VanDijk, Eikelenboom, and Hoving‐Bolink (2000). The parameters (L^*^ lightness, a^*^ redness and b^*^ yellowness) of meat colour were measured via a Minolta Chroma Meter CR‐300 colorimeter (Minolta, Osaka, Japan). A D65 illuminant and 10° standard observer angle were used. Besides, the chroma (C^*^) and hue angle (H^*^) were calculated with the following formulas: C^*^=√(a^*2^ + b^*2^) and as H^*^=tan^−1^(b^*^/a^*^)·57.29. The chemical composition including moisture, crude ash, calcium, phosphorus, IMF and protein contents of LM were determined and presented as the weight percentage of wet muscle tissue based on the AOAC methods (2005). The cooking loss was determined according to the previous method (Boccard et al., 1981). The meat samples (3 × 1.5 × 1.5 cm^3^) were placed in polyethylene bags and were heated at 75°C in a thermostatic water‐bath to an internal temperature of 72°C. After cooling at room temperature, weight was measured and expressed as a percentage of the initial sample weight. Furthermore, cooked chops were cut to 1 × 1×3 cm^3^ to measure the tenderness using a Tensipresser (TTP‐50BXII, Taketomo Electric Corp., Tokyo, Japan) to evaluate meat tenderness and using an up and down motion to imitate the meat chewing action.

### 2.5 Fatty acid profile

Fatty acid profile in LM was detected as fatty acid methyl ester derivatives in a PerkinElmer gas chromatographer (GC‐2010) using a method described by O’Fallon, Busboom, Nelson, and Gaskins (2007). Samples were freeze‐dried, and frozen for lipid extraction and methylation. The meat samples were taken out and thawed at room temperature for 6 hr. The middle part of the meat samples was taken with a scalpel and the surface fat was removed, and the powder was put into a mortar and grind with liquid nitrogen, then the powder of the meat sample (1 g) was put into a 1.5 ml centrifuge tube. Then, 0.7 ml of 10 mol/L KOH solution and 5.3 ml of anhydrous methanol (analytically pure) were added to the centrifuge tube. After mixture, the samples were put into a constant temperature water bath pot 55℃ for 1.5 hr. During this period, the test tube was oscillated every 20 min for 5 s. After the end of the water bath, the centrifugal tube was taken out and cooled it under room temperature through tap water. Then, 0.5 ml of 12 moLH_2_SO_4_ solution was added to the centrifuge tube, take a 55℃ water bath for 1.5 hr to make the free fatty acid methylation. Finally, the 3 ml of n‐hexane was added to the centrifuge tube and shaken, then centrifuged at 3,000 g for 5 min. Using a 2 ml disposable syringe and organic phase filter membrane, the supernatant was filtered and put into a GC vial for following detection. Samples were injected using an AI/AS 3,000 auto sampler (Thermo Fisher Scientific, Milan, Italy). A GC capillary column (Forte; SGE, Ringwood, Australia), 60 m long and with an interior diameter of 0.25 mm and 0.25 μm film thickness was used for separation of fatty acid. The flow speed was 3.2 ml/min with helium as carrier gas. The methyl esters of fatty acids were quantified using undecanoic acid methyl ester as an internal standard, and the methyl‐standard of each fatty acid was used as an external standard to calculate a regression line. Peak identification was based on the fatty acid ester standards (Supelco, Product No. 18,919. Sigma Product No. D5679. Fluka Product No. 43,959). Fatty acid profiles were estimated from the chromatogram peak areas and were expressed as the percentage of identified total fatty acid methyl esters.

### 2.6 RNA isolation and real‐time quantitative PCR

Real‐time quantitative PCR was used to verify the relative abundance of fatty acid synthase (FAS), acetyl‐CoA carboxylase (ACC), sterol regulatory element‐binding protein 1 (SREBP‐1), stearoyl‐CoA desaturase (SCD), peroxisome proliferator‐activated receptor γ (PPARγ), lipoprotein lipase (LPL), heart fatty acid‐binding proteins (H‐FABP), carnitine palmitoyltransferase‐1 (CPT‐1) and hormone‐sensitive lipase (HSL) at the mRNA level. The cDNA was reversely transcribed from the extracted RNA, which was separated from LM samples, using cDNA Synthesis Kit (TaKaRa, Dalian, China) reference to the descriptions. qRT‐PCR was conducted via the SYBR Green Kit (Sangon Biotechnology, Shanghai, China) and CFX96 Touch™ Real‐Time PCR System (Bio‐Rad Inc.) according to the specifications. The primers information are shown in Table 2. The β‐actin expression was used to normalize the targeted mRNA levels. The expression levels of all genes were calculated by the 2^−ΔΔCt^ method. All samples were processed in triplicates. The mean threshold cycle of triplicates of each sample was used in the calculations.

**TABLE 2:** Primers used for qRT‐PCR

Genes	Primes	Product size (bp)	Accession no.	Annealed temperature(°C)
β‐actin	F: 5’‐GATCTGGCACCACACCTTCTAC−3’ R: 5’‐GATCTGGGTCATCTTCTCACG−3’	115	AY_141970	56.0
FAS	F: 5’‐GCAAAGTGGTCATTCAGGTACG−3’ R: 5’‐CCCAGTGATGATGTAGCTCTTG−3’	125	NM_001012669	58.3
SCD	F: 5’‐ACTGCGGTCCAAGTCGTT−3’ R: 5’‐ACCCAGACAGAGGAGACTAAA−3’	296	NM_173959	59.4
ACC	F: 5’‐AAGCAATGGATGAACCTTCTTC−3’ R: 5’‐GATGCCCAAGTCAGAGAGC−3’	197	NM_174224	58.3
SREBP−1	F: 5’‐TTGAATAAATCTGCCGTCTTG−3’ R: 5’‐CCACTTCCACCGCTGCTACT−3’	292	NM_001113302	56.3
HSL	F: 5’‐ACGAGCCTTACCTCAAGAGCTG−3’ R: 5’‐CAGCAGTAGGCATAGGAGCACTC−3’	124	NM_001080220	56.3
CPT−1	F: 5’‐GGTCAACAGCAACTACTACG−3’ R: 5’‐TGAACATCCTCTCCATCTGG−3’	188	NM_001034349	56.3
H‐FABP	F: 5’‐GACCAAGCCTACCACAATCATC−3’ R: 5’‐GACTTTCCTGTCATCTGCTGTG−3’	136	BC_102153	59.4
PPARγ	F: 5’‐GACTTCTCCAGCATTTCCACTC−3’ R: 5’‐GGGATACAGGCTCCACTTTGAT−3’	133	AY_179866	59.4
LPL	F: 5’‐CTGGACGGTGACAGGAATGTAT−3’ R: 5’‐CAGACACTGGATAATGCTGCTG−3’	131	NM_001075120	59.4

Note

FAS, fatty acid synthase; SCD, stearoyl‐CoA desaturase; ACC, acetyl‐CoA carboxylase; SREBP‐1, sterol regulatory element‐binding transcription factor 1; HSL, hormone‐sensitive lipase; CPT‐1, carnitine palmitoyltransferase 1; H‐FABP, heart fatty‐acid‐binding protein; PPARγ, nuclear receptor peroxisome proliferator‐activated receptor gamma; LPL, lipoprotein lipase.

### 2.7 Statistical analysis

The data for analysis were used general linear model procedure of the SAS (version 9.4; SAS Institute, Inc.) software. The model used for the analysis was y_ij_ = μ + t_i_ + e_i_, where y represented the dependent variable, μ represented the population mean for the variable, t_i_ represented the fixed effect of diet treatments (diets = 3) and e_ij_ represented the random error associated with the observation ij. The sample size of growth performance was 5, and the sample size of other data was 6. The Tukey–Kramer procedure was used to perform multiple comparisons. Results were presented as least square means with their standard error of the mean. Significance was declared at *p* < .05.

## 3 RESULTS

### 3.1 Growth performance

Table 3 shows the effects of dietary energy concentration on growth performance of yaks. With the increase in dietary energy concentration, the final BW and ADG significantly increased (*p* < .05), while the F/G markedly reduced (*p* < .05). Compared to LE group, the final BW of ME and HE group increased by 10.90% and 45.16%, and the ADG increased by 55.70% and 168.51%, respectively.

**TABLE 3:** Effects of dietary energy concentration on growth performance of yaks

Items	Treatments^1^			*SEM*	*p* value
	LE	ME	HE		
Initial BW^2^ (kg)	111.56	108.30	119.47	3.841	.41
Final BW^2^ (kg)	145.99^b^	161.91^b^	211.92^a^	6.787	< .01
ADG^3^ (g/d)	286.92^c^	446.75^b^	770.42^a^	43.880	< .01
ADFI^4^ (kg/d）	3.86	3.83	3.93	0.037	.47
F/G^5^	14.10^a^	9.43^b^	5.25^c^	0.822	< .01

^a,b,c^Means within a row with different superscript letters differ at the *p* < .05 level.

^1^ LE, low energy; ME, medium energy; HE, high energy.

^2^ BW, body weight.

^3^ ADFI, average daily feed intake.

^4^ ADG, average daily gain.

^5^ F/G, the ratio of feed intake to gain.

### 3.2 Carcass traits

Effects of dietary energy concentration on carcass traits of yaks are presented in Table 4. Data pertaining to the liveweight prior to slaughter, carcass weight, dressing percentage, meat percentage, back fat thickness and loin muscle area were all improved significantly (*p* < .05) by increasing dietary energy concentration. Compared with the LE group, the dressing percentage of ME and HE group increased by 3.50% and 9.83%, while the back fat thickness of the ME and HE groups increased by 168.75% and 481.75%, respectively.

**TABLE 4:** Effects of dietary energy concentration on carcass traits of yaks

Items	Treatments^1^			*SEM*	*p* value
	LE	ME	HE		
Before slaughter liveweight (kg)	143.60^c^	165.10^b^	207.93^a^	7.351	< .01
Carcass weight (kg)	64.25^c^	76.58^b^	105.83^a^	4.518	< .01
Dressing percentage (%)	44.80^c^	46.37^b^	50.93^a^	0.667	< .01
Meat percentage (%)	34.61^c^	37.38^b^	42.64^a^	0.861	< .01
Back fat thickness (cm)	0.16^c^	0.43^b^	0.83^a^	0.072	< .01
Loin muscle area (cm^2^)	30.03^c^	32.83^b^	35.69^a^	0.566	< .01

^a,b,c^Means within a row with different superscript letters differ at the *p* < .05 level.

^1^ LE, low energy; ME, medium energy; HE, high energy.

### 3.3 Primal cuts

Effects of dietary energy concentration on primal cuts of yaks are shown in Table 5. The ribeye, high rib, outside flat, eyeround, topside and knuckle weight of the HE group were significantly higher (*p* < .05) than those in the LE group and ME group, but no significant difference (*p* > .05) was noted between the LE group and ME group. The striplion, brisket, shank and shoulder chops weight markedly increased (*p* < .05) with the dietary energy level increased. Numerical difference (*p* > .05) was noted in the tenderlion and chuck tender weight among all groups.

**TABLE 5:** Effects of dietary energy concentration on primal cuts weight of yaks

Items	Treatments^1^			*SEM*	*p* value
	LE	ME	HE		
Striplion (kg)	1.73^c^	2.27^b^	2.97^a^	0.127	< .01
Ribeye (kg)	2.13^b^	2.57^b^	4.10^a^	0.237	< .01
High rib (kg)	3.43^b^	4.2^b^	5.73^a^	0.278	< .01
Brisket (kg)	1.82^c^	2.20^b^	2.62^a^	0.094	< .01
Tenderlion (kg)	1.30	1.50	1.57	0.088	.23
Shank (kg)	5.98^c^	6.87^b^	8.97^a^	0.326	< .01
Chuck tender (kg)	1.10	1.17	1.33	0.063	.13
Shoulder chops (kg)	3.50^c^	4.32^b^	5.38^a^	0.195	< .01
Outside flat (kg)	3.03^b^	3.27^b^	4.13^a^	0.134	< .01
Eyeround (kg)	0.83^b^	1.03^b^	1.28^a^	0.065	< .01
Topside (kg)	4.43^b^	4.70^b^	5.90^a^	0.205	< .01
Kunckle (kg)	3.37^b^	3.40^b^	4.27^a^	0.141	< .01

^a,b,c^Means within a row with different superscript letters differ at the *p* < .05 level.

^1^ LE, low energy; ME, medium energy; HE, high energy.

### 3.4 Meat quality

As shown in Table 6, the increased dietary energy concentration resulted in a significant decrease (*p* < .05) of cooking loss, driage and shearing force. The shearing force of the ME and HE groups decreased by 17.57% and 30.48%, respectively, compared to LE group. As far as the chemical composition of the yak meat was concerned, the IMF content of the ME and HE groups increased (*p* < .05) by 36.62% and 76.76% in comparison with the LE group. Moreover, the moisture content observably decreased (*p* < .05) as the dietary energy level increased. No significant influence (*p* > .05) of dietary energy concentration on pH_45min_, pH_24h_, meat colour parameters, protein, crude ash, calcium and phosphorus content was detected.

**TABLE 6:** Effects of dietary energy concentration on meat quality of yaks

Items	Treatments^1^			*SEM*	*p* value
	LE	ME	HE		
pH_45min_ ^2^	6.62	6.54	6.60	0.026	.82
pH_24h_ ^3^	5.76	5.80	5.73	0.058	.83
Cooking loss (%)	29.70^a^	27.61^b^	26.45^c^	0.336	< .01
Driage (%)	25.34^a^	24.03^b^	22.31^c^	0.311	< .01
Shearing force (kg/cm^2^)	6.43^a^	5.30^b^	4.47^c^	0.204	< .01
Colour parameters					
Lightness (L*)	33.20	34.30	35.49	0.468	.40
Redness (a*)	17.58	19.02	20.64	0.236	.53
Yellowness (b*)	7.27	9.16	9.70	0.130	.65
Chroma (C*)	21.48	21.33	21.87	0.215	.47
Hue angle (H*)	21.84	22.59	21.81	0.435	.98
Chemical composition					
Intramuscular fat (%)	1.42^c^	1.94^b^	2.51^a^	0.110	< .01
Protein (%)	22.80	22.70	22.32	0.128	.30
Moisture (%)	74.84^a^	73.93^b^	72.78^c^	0.227	< .01
Crude ash (%)	1.11	1.14	1.15	0.041	.95
Calcium (%)	0.04	0.05	0.04	0.002	.42
Phosphorus (%)	0.23	0.23	0.22	0.008	.84

^a,b,c^Means within a row with different superscript letters differ at the *p* < .05 level.

^1^ LE, low energy; ME, medium energy; HE, high energy.

^2^ pH measured 45 min after slaughter.

^3^ pH measured 24 hr after slaughter.

### 3.5 Fatty acid profile

Table 7 shows the fatty acid profile of the LM of different dietary treatment groups. Higher (*p* < .05) proportions of C16:0 were found in lower energy concentration diets. However, the proportions of both C18:2 and C20:4 were increased (*p* < .05) with an increase to dietary energy concentration. The polyunsaturated fatty acid (PUFA) content increased at the expense of the saturated fatty acids (SFA) content with the dietary energy level increased (*p* < .05), though no significant (*p* > .05) effect on dietary energy concentration was detected on monounsaturated fatty acid (MUFA) proportions.

**TABLE 7:** Effects of dietary energy concentration on fatty acid profile of the *longissimus thoracis* of yaks

Items	Treatments^1^			*SEM*	*p* value
	LE	ME	HE		
∑SFA^2^ (%)	47.81^a^	47.35^b^	46.79^c^	0.109	< .01
C13:0 (%)	0.30	0.30	0.30	0.005	.81
C14:0 (%)	2.01	2.00	1.99	0.009	.38
C15:0 (%)	0.40	0.39	0.41	0.007	.40
C16:0 (%)	23.89^a^	23.51^b^	22.92^c^	0.102	< .01
C17:0 (%)	1.22	1.22	1.22	0.013	.57
C18:0 (%)	19.26	19.21	19.21	0.014	.20
C20:0 (%)	0.47	0.45	0.47	0.004	.77
C22:0 (%)	0.17	0.16	0.17	0.003	.64
C24:0 (%)	0.10	0.11	0.11	0.002	.60
∑MUFA^3^ (%)	43.32	43.39	43.49	0.039	.08
C16:1 (%)	3.70	3.71	3.69	0.013	.76
C18:1 (%)	39.62	39.69	39.80	0.045	.11
∑PUFA^4^ (%)	8.84^c^	9.23^b^	9.71^a^	0.093	< .01
C18:2 (%)	4.21^c^	4.45^b^	4.64^a^	0.055	< .01
C18:3 (%)	0.14	0.15	0.14	0.003	.84
C20:4 (%)	3.54^b^	3.66^b^	3.97^a^	0.054	< .01
C20:5 (%)	0.94	0.95	0.94	0.006	.67
C22:5 (%)	0.03	0.02	0.03	0.002	1.00

^a,b,c^Means within a row with different superscript letters differ at the *p* < .05 level.

^1^ LE, low energy; ME, medium energy; HE, high energy.

^2^ SFA, saturated fatty acid.

^3^ MUFA, monounsaturated fatty acid.

^4^ PUFA, polyunsaturated fatty acid.

### 3.6 Gene expression

**FIGURE 1:**
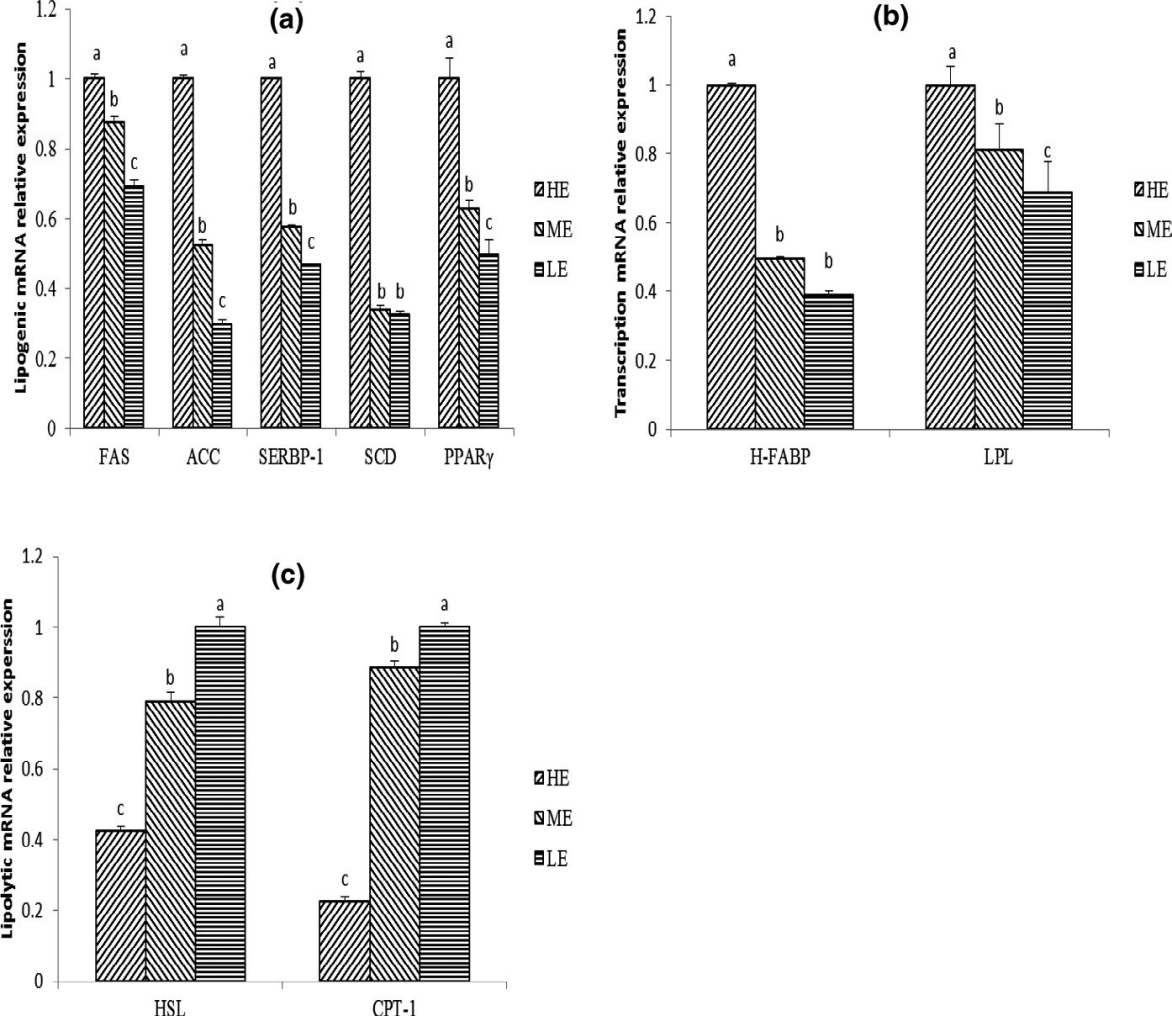
Effects of dietary energy concentration on gene expression in *longissimus thoracis* of yaks. (a) Expression levels of lipogenic genes. (b) Expression levels of transcription genes. (c) Expression levels of lipolytic genes. LE, low energy; ME, medium energy; HE, high energy; FAS, fatty acid synthase; SCD, stearoyl‐CoA desaturase; ACC, acetyl‐CoA carboxylase; SREBF‐1, sterol regulatory element‐binding transcription factor 1; PPARγ, nuclear receptor peroxisome proliferator‐activated receptor gamma; HSL, hormone‐sensitive lipase; CPT‐1, carnitine palmitoyltransferase 1; H‐FABP, heart fatty‐acid‐binding protein; LPL, lipoprotein lipase. Means within different superscript letters differ at the *p* < .05 level

Figure 1 shows that as dietary energy concentration increased, the mRNA expression levels of FAS, ACC, SREBP‐1, SCD, PPARγ, LPL and H‐FABP significantly increased (*p* < .05), while the HSL and CPT‐1 decreased (*p* < .05).

## 4 DISCUSSION

### 4.1 Growth performance

Dietary energy concentration determines the consumption of feed as well as the supply of protein and other nutrients, and has an important role in animals’ growth production (Jobgen, Fried, Fu, Meininger, & Wu, 2006; Lei, Yan, Kim, & Kim, 2018). To our knowledge, this was the first study designed to determine the effects of dietary energy concentration on yaks’ growth performance and meat quality that were raised indoors. In our present study, when the dietary concentration increased from 3.72 to 5.32 MJ/kg, the ADG of yaks increased linearly to 770.42 g/d, which was comparable with ordinary yellow cattle. Recent research has reported that steers fed 80% and 60% of the maintenance energy requirement exhibit a decreased BW gain (Lima, Sucupira, & Ortolani, 2014). Peng et al. (2012) also have found that there are an increased ADG and a decreased F/G in beef cattle when dietary energy density increased. Similarly, Dong et al. (2006) reported a linear increase in daily liveweight gain of yaks when dietary metabolizable energy increased from 8.00 to 10.26 MJ/kg. This results prove again that dietary energy concentration plays an important role in the growth production of yaks in the cold season. In addition, the ADG of the ME group was 447 g/d, which was less than our expectation (800 g/d). Therefore, based on our results, it could be speculated that yaks have higher energy requirements for maintenance and growth in comparison to ordinary yellow cattle.

### 4.2 Carcass traits

Previous study studied the carcass characteristics of Jiulong‐yak (a bigger physique yak variety) at the end of the warm season (October) and the data showed that 2.5 ~ 3.5 years old grazing male yak had a slaughter weight of 218.4 kg (Zi et al., 2004). In the present study, compared to the LE and ME groups, the 3 years old Qinghai plateau male yaks in the HE group had a higher slaughter weight, dressing percentage and a higher backfat thickness, which suggested that yaks raised by barn feeding resulted in promoted carcass fat deposition and improved carcass traits in cold season. The previous studies reported that improving dietary energy concentrations or energy intake could increase the carcass weight, dressing percentage and back fat thickness, but reduce the loin eye area (Prior, Kohlmeier, Cundiff, Dikeman, & Crouse, 1977; Zhang et al., 2014), which were basically in line with our study. Hence, it might be a beneficial means of manipulating carcass characteristic of yaks by adjusting dietary energy concentrations in the barn feeding conditions. However, we found that the loin muscle area was enhanced with the increase in energy concentration, which was in agreement with the results of Cromwell et al. (1978). Besides, a larger loin muscle area means more primal cuts.

### 4.3 Primal cuts

The primal cuts represent most of the commercial value of the carcass. Some high‐grade markets require a certain weight for each cut. The weight of almost all primal cuts in the current study was higher in the HE group than that in the LE and ME groups, suggesting that high dietary energy concentration contributed to the higher production of the primal cuts. This results were in line with previous research (Li, Wang, He, & Cao, 2014). Li et al. (2014) have reported that high‐energy density diet improves yields of top and medium top grade commercial meat cuts of the Chinese Xiangxi yellow cattle. It might be speculated that yaks on the Qinghai‐Tibetan plateau are usually under the energy deficient conditions, which hinder the growth potential of yaks. In the present study, the primal cuts weight increased in a linear manner along with the increase in dietary energy concentration, which indicated that the energy density of the HE group did not reach the yaks maximum potential growth, and the optimal energy requirement of yaks in cold season warranted further investigations. The available information relating to the effects of dietary concentration on the proportion of primal cuts of yak carcass is rare, and no comparisons could be made. In contrast, Suarezbelloch, Sanz, Joy, and Latorre (2013) found a decreased percentage of ham and loin in pigs as the dietary NE density increased from 2,280 to 2,420 Kcal/kg during the finishing period. In addition, Cámara, Berrocoso, Sánchez, López‐Bote, and Mateos (2014) reported similar results for gilts and boars. This might be impacted by the increased fat deposition of pigs during the finishing period along with the increase in dietary energy concentration. The yaks in present study were slaughtered before a large amount of fat deposited, only muscle development was strengthened.

### 4.4 Meat quality

Higher dietary energy intake has positive effects on the meat quality. In the present experiment, increasing dietary energy concentration significantly decreased yak cooking loss, driage, shearing force and increased yaks intramuscular fat content. This findings were mirrored by the results of Moloney and Drennan (2013) and Manni, Rinne, and Huhtanen (2013), who reported that increased dietary energy intake improved meat quality traits of finishing beef cattle. In addition, Zeng, Yu, Mao, and Chen (2012) reported that increasing the dietary digestible energy concentration from 3.2 to 3.8 Mcal/kg significantly decreased the Rongchang piglets cooking loss and shearing force. However, some studies have reported that dietary energy concentration has no significant effect on the meat quality traits of goats and pigs (Abdullah & Musallam, 2007; Matthews et al., 2003). This might be due to the differences among the experimental dietary energy concentration and animal growth stage. Previous study defined tender, intermediate and tough steaks as < 3.0 kg/cm^2^, between 3.0 and 4.6 kg/cm^2^, and > 4.6 kg/cm^2^ of the shearing force, respectively (Li et al., 2014). Based on these data, yak beef under the traditional farming system may be regarded as tough steak. In the current study, the shearing force decreased from 6.43 to 4.47 kg/cm^2^ with the dietary Nemf concentration increasing from 3.72 to 5.32 MJ/kg. This means that the yak beef of the HE group meet the standard of intermediate steak. The decreased shearing force might have resulted from an increase in the IMF content. On the other hand, it was regarded that IMF affects the modification of muscle fibre condition, the composition and content of connective tissue, and configuration of protease in muscle, which can affect muscle tenderness (Gerbens et al., 2001; Joo, Kim, Hwang, & Ryu, 2013).

### 4.5 Fatty acid profile

The fatty acid profile of meat has an important effect on meat quality, sensory characteristics, consumer acceptance and human health. Compared to the non‐ruminant animals, in ruminants, the MUFA and PUFA suffer a ruminal biohydrogenation process, which is responsible for the saturation of the dietary fatty acids. Therefore, ruminants do not deposit tissue fatty acids in proportion to dietary lipid composition (Edwards et al., 2012). In spite of this, efforts were still made to obtain a healthier fat acid profile in ruminants. In our study, the PUFA content increased at the expense of the SFA content, with the highest dietary energy concentration, while no significant effect was seen on the diet energy concentration on MUFA proportions. Similar results were also found in yellow cattle (Li et al., 2014). It is believed that high dietary energy concentration is more conducive to limit dietary fatty acid saturation by rumen microbes. Conversely, Smet, Webb, Claeys, Uytterhaegen, and Demeyer (2000) have reported that bulls fed higher energy diets have higher proportions of C16: 0 (but lower C18:2 and C20:4). It might be attributed, to some extent, to the difference of dietary fatty acid composition between the two experiments.

### 4.6 Gene expression

IMF content is an important economic characteristic of beef products. In the current study, the IMF content increased as the dietary energy concentration increased. This was in accordance with Smet et al. (2000), who reported an increased IMF in Belgian blue bulls fed with high‐energy diets, which was much more higher than our data (9.9% VS 2.51%). An attempt was made to elucidate the underlying mechanisms manipulating fat deposition. The expression level of genes responsible for lipid metabolism, including lipogenesis, lipolysis and fatty acid transport was all examined.

As a nuclear transcription factor, PPARγ has been demonstrated to regulate the expression of several genes encoding proteins involved in adipocyte differentiation and fat deposition (Moisá et al., 2014). SREBP‐1 is a key transcription factor in the regulation of the expression of lipogenic genes, including FAS, ACC and SCD (Doran et al., 2006). FAS, a key enzyme, catalyses the entire pathway of palmitate synthesis from malonyl‐CoA in mammals (Smith, Witkowski, & Joshi, 2003). ACC is a key rate‐limiting enzyme responsible for de novo fatty acid biosynthesis (Brownsey, Boone, Elliott, Kulpa, & Lee, 2006). SCD is a key enzyme responsible for triglyceride syntheses by providing a better accessible pool of monounsaturated fatty acids (Schmid, Collomb, Sieber, & Bee, 2006). Our data showed that the mRNA expression of the above lipogenic genes increased as the dietary energy concentration increased. Consistent with our study, Graugnard et al. (2009) have found that cattle fed high‐starch diets have higher mRNA expressions of PPARγ, SREBP‐1, ACC, FAS and SCD in Angus and Angus × Simmental compared with that of those fed with low‐starch diets. In addition, another study obtained a lower mRNA expression of FAS and SCD in adipose tissue of energy‐restricted obese women (Dahlman et al., 2005). Altogether, this data suggested that an increase in energy intake promotes the expression of lipogenic genes. The up‐regulated expression of these lipogenic genes means there is an increased capacity for de novo synthesis of FFA, leading to an increase in IMF accumulation.

Besides lipogenic capacity, the IMF accumulation is also associated with fatty acid transport capacity in adipose tissue. LPL acts as the rate‐limiting enzyme in the hydrolysis of triglycerides that exists in circulating chylomicrons and very‐low‐density lipoprotein, and has been described as the ‘metabolic gatekeeper’ (Wang et al., 2009). H‐FABP is a member of the family of intracellular fatty acid‐binding proteins involved in the intracellular targeting of fatty acids and facilitates the transport of fatty acids from the membrane to the sites of fatty acid oxidation or esterification into total triglycerides or phospholipids (Chmurzyńska, 2006). Our study showed that the mRNA expression levels of LPL and H‐FABP were increased following the increase in dietary energy content. These results were in line with previous investigations (Peng et al., 2012; Zhang et al., 2014), who reported an increased mRNA expression of LPL in the LM of finishing cattle when dietary energy levels were enhanced. Based on the above results, we can speculate that high dietary energy concentration can up‐regulate the expression of fatty acid transport genes in IMF and result in an increased IMF deposition.

Finally, the accumulation of IMF is a result of lipogenesis and lipolysis. HSL is a key enzyme for fatty acid mobilization in adipocytes and skeletal muscles, and is the rate‐limiting enzyme in triglyceride degradation in all situations (Holm, Østerlund, Laurell, & Contreras, 2000). HSL activity has been proven to be negatively correlated with IMF content in the LM of Wagyu hybrid cattle, and may have a predictive value for assessing the potential of cattle to deposit IMF (Kazala et al., 2003). CPT‐1 is a rate‐limiting enzyme in triacylglycerol catabolism and is responsible for importing esters from fatty acids into the mitochondria for β‐oxidation (DeBerardinis, Lum, & Thompson, 2006). In the current study, the mRNA expression of HSL and CPT‐1 was decreased as the dietary energy concentration increased, which suggested that the triacylglycerol breakdown and fatty acid oxidation in the LM were inhibited. These results were consistent with the results of Zhang, Liu, Cheng, and Song (2012), who found that in the LM, low energy of diet could increase the HSL mRNA levels as compared to those of the middle and high‐energy diets. These results indicated that the increased IMF content along with the enhanced dietary energy was due to the reduced expression of lipolytic genes.

## 5 CONCLUSION

Based on the data we obtained, we concluded that high dietary energy concentrations can significantly improve growth performance, meat production and meat quality of finishing yaks raised by barn feeding. In addition, the observations highlighted the underlying molecular mechanisms of IMF deposition and indicated that the increase in IMF accumulation, associated with dietary energy concentration, related mainly to up‐regulation of intramuscular lipogenic genes expression and fatty acid transport genes expression, as well as the down‐regulation of lipolytic gene expression. This suggests to us that yaks beef products could be manipulated via nutritional management. Finally, according to our results, the yaks have higher energy requirements for maintenance and growth.

## 6 ANIMAL WELFARE STATEMENT

The experimental protocol used in the present study was approved by the Animal Policy and Welfare Committee of the Agricultural Research Organization of Sichuan Province, China, and was in accordance with the guidelines of the Animal Care and Ethical Committee of the Sichuan Agricultural University.

## CONFLICT OF INTEREST

The authors declare that there is no conflicts of interest.

## AUTHORS CONTRIBUTION

K.K.: Data curation; Investigation; Methodology; Software; Writing‐original draft; Writing‐review & editing. J. M.: Data curation; Investigation; Methodology; Software; Writing‐original draft; Writing‐review & editing. H.W.: Data curation; Investigation; Writing‐review & editing. Z.W.: Conceptualization; Funding acquisition; Project administration; Supervision; Validation; Writing‐review & editing. Q.P.: Formal analysis; Writing‐review & editing. R.H.: Data curation; Writing‐review & editing. Huawei Zou: Writing‐review & editing. SB: Conceptualization; Supervision; Writing‐review & editing. WZ: Supervision; Writing‐review & editing. B.S.: Conceptualization; Supervision; Writing‐review & editing. The study was designed by Zhisheng Wang, Shanke Bao, Wenhua Zhang and Baozhong Sun. Kun Kang, Jian Ma and Hongze Wang performed this study, and Kun Kang, Jian Ma, Rui Hu and Huawei Zou determined the samples. Kun Kang and Jian Ma analyzed the data and wrote the original paper. Quanhui Peng revised the paper, and all authors read and approved the final manuscript.

## Data Availability

The data that support the findings of this study are available from the corresponding author upon reasonable request.
